# The Promotion of Hearing Health through Wikipedia Campaigns: Article Quality and Reach Assessment

**DOI:** 10.3390/healthcare11111572

**Published:** 2023-05-27

**Authors:** Alexandre Alberto Pascotto Montilha, Thais Catalani Morata, Daiana Ávila Flor, Maria Aparecida Andrade Moreira Machado, Fabrício Augusto Menegon, Fernanda Zucki

**Affiliations:** 1 Educational Technology Section, Bauru School of Dentistry, University of Sao Paulo, Bauru 17012-901, SP, Brazil; 2 Noise and Bioacoustics Team, National Institute for Occupational Safety and Health, Centers for Disease Control and Prevention, Cincinnati, OH 45226, USA; tmorata@cdc.gov; 3 Department of Speech-Language Pathology and Audiology, Federal University of Santa Catarina, Florianopolis 88040-370, SC, Brazil; daianaavilaflor@gmail.com (D.Á.F.); fernanda.zucki@ufsc.br (F.Z.); 4 Department of Pediatric Dentistry, Orthodontics and Public Health, Bauru School of Dentistry, University of Sao Paulo, Bauru 17012-901, SP, Brazil; mmachado@fob.usp.br; 5 Department of Public Health, Federal University of Santa Catarina, Florianopolis 88040-370, SC, Brazil; fabricio.menegon@ufsc.br

**Keywords:** health promotion, encyclopedia, crowdsourcing, educational technology, health campaigns, audiology

## Abstract

This case study examined the feasibility, reach, and potential impact of using Wikipedia as a tool for hearing health promotion. Activities involved editing existing Portuguese-language Wikipedia hearing health articles, as well as translating English-language hearing health articles to Portuguese during the *Wiki4WorldHearingDay2019* and *Wiki4YearOfSound2020* online campaigns. The Wikipedia efforts that took place in Brazil were carried out by 10 volunteer undergraduate students in Speech-Language Pathology and Audiology at the Federal University of Santa Catarina, in Brazil. Among new and existing Wikipedia articles, the group edited 37 articles, which attracted more than 220,000 views during the set tracking period. Students were responsible for 60% of the Portuguese-language edits during the *Wiki4WorldHearingDay2019* campaign and more than 90% of the Portuguese-language edits during the first half of the *Wiki4YearOfSound2020* campaign. Moreover, the quality indexes for pages either created or edited were improved in all situations by registering an increase rate ranging from 33% to 100%. Wikipedia-centered activities expanded the availability of quality scientific content, written in plain language, to the public. Students worked together in order to select topics, assess existing information, validate it, create new content, and share information—steps that contributed to the mission of health promotion and knowledge dissemination for the benefit of society.

## 1. Introduction

The lack of quality information about health conditions and existent health services is considered an obstacle to promoting public health. This challenge is demonstrated with hearing health [1], considering the several year gap between the first signs of hearing difficulties and the subsequent search for healthcare [2,3,4,5,6]. New technologies, expanded access to telehealth, and open communication are needed to change this scenario [7]. Information available in simple and accessible language can help people understand and implement health information into their daily lives. Access to free information is recognized by the Centers for Disease Control and Prevention as a necessary step in promoting health in general [8].

We selected Wikipedia as the platform to explore new science communication efforts. Wikipedia is seen as a significant digital platform for sharing information because of its open access mission and inclusive and democratic policies and practices [9]. Wikipedia.com holds seventh place among the most visited websites around the world, with 400 million unique visitors in 2018, accounting for 7.8 million pageviews per month [10]. In 2013, a survey showed that Wikipedia’s medical content consisted of more than 155,000 articles and one billion bytes of text in over 255 languages, and the number of views that year reached an impressive number of 4.9 billion. As a result, Wikipedia can be considered the most viewed medical resource worldwide [11], and its academic studies are quickly proliferating [12].

The largest Wikipedia is the English Wikipedia. In September 2021, it had more than 6 million articles and 42 million registered users, with about 123,000 of those being regularly active in the platform [13]. The Portuguese Wikipedia had just over one million pages of encyclopedic content and more than two million registered users, of which about nine thousand are active [14]. In 2021, among the different language versions of Wikipedia, it ranked 15th in the number of articles created [15]. Health-related content is the most visited on the platform in Portuguese, ranking prominently in Google’s search results [16].

Scientific content in Wikipedia is unevenly developed. Thus, the Wikimedia Foundation (WMF), which is the nonprofit organization that hosts Wikipedia and its sister projects, has partnered with scientific academic institutions and public health agencies to expand and improve the coverage of scientific topics [17]. The National Institute for Occupational Safety and Health (NIOSH) is one of the research agencies engaged in this effort. NIOSH developed and now manages WikiProject Occupational Safety and Health, which is a WikiProject that improves occupational safety and health content on Wikipedia. NIOSH also participates in classroom programs aimed at broadening and improving Wikipedia occupational health content and health promotion campaign organization [17,18].

Expanding aspects of scientific practice, such as the transparency, productivity, and reproducibility of scientific research, is crucial to provide relevant information to assist the public in making informed health decisions. This is in addition to having a favorable impact on the community and science-based policies [19,20]. In this sense, Wikipedia activities address two important scenarios: health promotion and scientific dissemination. Its activities promote new and dynamic processes for sharing information. In addition, Wikipedia publishes topics of interest in certain areas, develops and/or improves scientific communication skills, and improves digital literacy, which is a person’s ability to locate, assess, and articulate information via digital media. These activities can be measured in different ways, including the number of times a Wikipedia page is visited and interacted with by other editors on the platform [21,22].

This study was conceptualized from the experiences and accomplishments of leading health organizations such as Cochrane, the World Health Organization, Cancer Research UK, and NIOSH. Each institution developed customized strategies to integrate content into Wikipedia. The Participatory Action Research [23] approach inspired some of the methods used in this case study and involved the active participation of the students of Speech-Language Pathology and Audiology in health education (Audiology Study Group). Evidence suggests that expanding health content in Wikimedia’s public digital knowledge archives can increase and expedite its reach to the general public [11,16].

This study aimed to examine the feasibility, reach, and impact of a specific strategy for hearing health promotion within a formal education setting in coordination with global awareness-raising campaigns on Wikipedia and used a set of specific metric tools.

## 2. Materials and Methods

### 2.1. Feasibility of the Proposed Model: Training of the Student Participants

In our study, undergraduate students became coauthors in a collective production of knowledge. They learned how to edit encyclopedia pages and then put into practice their new knowledge by editing Wikipedia. Their participation was voluntary, as well as their choice of Wikipedia account names. They were informed of the public and transparent nature of the assignment and the plans to analyze the experience in a feasibility/case study.

A formal training process was developed and implemented. As participation was voluntary and did not have any formal grading or other performance evaluation, the main indicator of feasibility was retention. Attention, participation, and effort were also indicators of the viability of the approach. The project was publicized among the students through virtual channels, and interested parties signed up. Participants were eligible to participate if they could dedicate a minimum of two hours a week, which included participating in project meetings.

Ten undergraduate students volunteered to participate in the first proposed activity, which was part of the *Wiki4WorldHearingDay2019* campaign. They were invited to contribute to a second campaign, *Wiki4YearOfSound2020*, and eight accepted. These two campaigns were selected because of the prominence of the organizing institutions and their common objectives to expand outreach and increase awareness among the widest possible audience. We noted that the campaigns also give students not only the motivation but also the opportunity to exercise new technical skills to expand the scientific content in hearing health while being able to measure the impact of their activities with objective metrics.

Study activities involving Wikipedia began in January 2019 and included the following: (1) locating and evaluating specific content on the intersection of audiology and environmental and occupational health; (2) discussing scientific evidence in these topic areas; (3) engaging in the health education process; and (4) introducing students to a public-directed scientific writing experience. In a partnership with researchers from NIOSH, the agency overseeing the two Wikipedia global online campaigns, the group focused on topics related to the promotion of hearing health.

Students took an 8 h training course offered by experienced Wikipedia writers and editors, called Wikipedians, from the Federal University of Santa Catarina. They used educational materials and tutorials developed by the Wiki Movement Brazil User Group (WMB) [24], the Wikipedia Education Program [25], and other collaborators. Participants learned about tools that are available for translating from Wikipedia: Translation [26] and from the training library of Wiki Education. This Wikipedia training covered the theoretical framework of the platform while hands-on experience demonstrated the processes for editing, translating, and developing content [27]. In addition, the Wikipedia Crowdsourcing Group from the University of Sao Paulo held online workshops and technical support training sessions throughout the effort. The main objective of the training was to show the students how to locate and evaluate sources for their verifiability, to discover associated content gaps in Wikipedia gaps, and to improve the content on the platform.

After the training was completed, students individually carried out writing assignments, which were tracked as part of the *Wiki4WorldHearingDay2019* campaign. The campaign platform (available in 17 languages) had guidance on how to identify topics and improve or translate existing Wikipedia articles. This campaign was cohosted by the World Health Organization (WHO) and several professional organizations. A project coordinator provided online weekly guidance to students.

### 2.2. Analysis Methods for Reach and Quality of Student Contributions

The number of pageviews before these activities began, as well as changes in pageview trends afterward, was tracked by a set of online tools developed, maintained, and hosted by the Wikimedia Cloud Services team and volunteers [28]. The tracking tools used were Outreach Dashboard, Pageviews Analysis [29], and xTools [30]. The tools, which were hosted at Wikimedia Toolforge Services, performed analytics, administered bots, and ran specific web services, as well as created other tools [31]. The “number of views” metric provided information on the interest from Wikipedia readers on a specific subtopic [11,32].

The Outreach Dashboard was used to track students’ activities and determine the amount of content both in kilobytes (kB) and in number of words added to each page during the campaigns. The dashboard also provided the number of articles created and edited, along with the number of citations and other media files added. The Pageviews Analysis tool and xTools were both applied to the precise number of pageviews in a given period: 30 days before activities began and 30 days after they ended.

**Figure 1:**
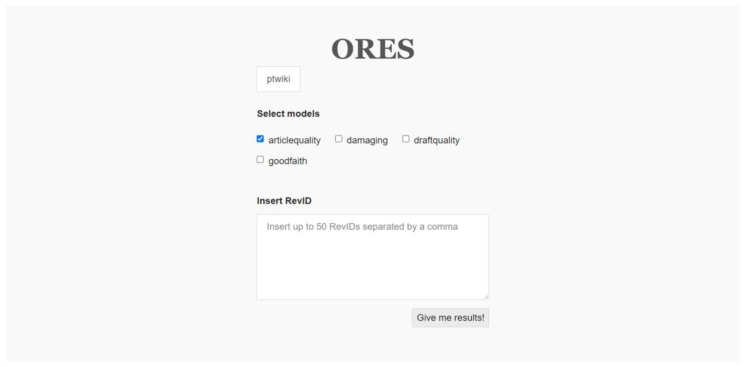
The ORES User Interface (UI) tool and the *articlequality* model. The tool is hosted at https://ores.wikimedia.org/ui/ (accessed on 6 April 2023).

Beyond the quantitative analysis of pageviews, an evaluation criterion for both improved and translated contents was provided. An online tool that uses semantic data tracked the evolution of content through article quality assessments based on Objective Revision Evaluation Service (ORES) technology [33], developed and maintained by the Wikimedia Machine Learning team (shown in Figure 1). Essentially, it is an artificial intelligence service that runs on the backend and aims to provide machine learning features for an ecosystem of tools available on Wikimedia projects [34]. These tools make it possible to automate critical and time-consuming work in the context of Wikimedia projects [35]. Their most common applications include counter-vandalism tasks and quality predictions of new edits and articles. For the latter, ORES adopts a combination of open data and open-source machine learning algorithms to train models and create scores, which may vary depending on the Wikipedia project and its respective quality grading scheme. ORES scores help predict the quality of edits as they are made to the pages, as well as the quality of an article based on each given history revision (RevID).

On English Wikipedia, the quality assessments are made using a grading scheme aimed to determine the distribution quality of an article on a particular topic within a WikiProject, which is defined as the Wikipedia 1.0 Project. Usually, these quality assessments are carried out by editors who manually tag pages of articles. Then these tags are automatically collected by a bot that generates logs and statistics in the form of matrices of quality or relevance [36,37]. On the Portuguese Wikipedia, an equivalent grading scheme is composed of six indexes (ranging from 1 to 6), with the highest being the best quality rating [38,39,40,41,42,43,44,45].

For an article to be indexed as quality, the algorithm takes into account the semantic structures that indicate quality criteria and possible syntax or editing issues, both conditions affecting the final score [38]. Other criteria such as verifiability and the number of reliable sources determine the outcome [46]. Overall, verifiability, neutral point of view, compliance to the manual of style, presence of sections and images, and completeness are positively rated by the algorithm. The absence of reliable sources, internal or external links, a lack of neutrality (or a disrespect of other content core policies, such as those related to verifiability and original research), and a poor layout negatively affect the final score [46].

Thus, the *articlequality* model of the ORES UI tool was applied in two circumstances on each page:

1. To compare the index of quality after the last history revision prior to student activities to the last history revision after the student interventions to find the improved quality.
2. To obtain the index of quality after the last history revision, conducted right after the end of student activities (for the created/translated content).

In the case of translated pages, only the revision history was used to obtain the quality index. Then comparisons regarding the evolution of the indexes between the two forms of activities (content creation/translation vs. content improvement) were carried out to determine the effectiveness of each approach and its impact.

## 3. Results

### 3.1. Global Online Campaigns

Activities from the *Wiki4WorldHearingDay2019* campaign were tracked from 21 January 2019 through 31 March 2019. Overall, the results indicated that 74,000 words were contributed to 90 existing and 7 new Wikipedia articles, and 21 images were donated to the open access repository WikiCommons. These expanded (edited) and new articles received more than 2.8 million views during the 2-month tracking period. The most accessed English-language pages during the entire *Wiki4WorldHearingDay2019* campaign were hearing loss, tinnitus, otitis media, and noise-induced hearing loss. The Portuguese-language articles with the highest number of views were hearing and speech therapy, effects of noise pollution on health, solvents, noise-induced hearing loss, and occupational hearing loss. Four of these Portuguese-language articles were edited by study participants.

Since the beginning of the subsequent *Wiki4YearOfSound2020* campaign on 4 November 2019, overall, more than 1000 articles were created or expanded in several languages, including English, Portuguese, Spanish, Italian, Chinese, and French, in a 12-month period. At the end of 2020, the entire campaign had achieved more than 131 million views for those 1000+ articles. One must note, however, that because pages had been individually edited at different times throughout the campaign, the duration of the tracking period varied with the date of the edit (i.e., articles tracked early in the campaign have longer tracking periods).

### 3.2. Outcomes by the Brazilian Students’ Efforts as an Extension Activity: Quantitative Assessment

The student participants were responsible for 60% of the Portuguese-language edits for *Wiki4WorldHearingDay2019* campaign and more than 90% of the Portuguese-language edits for the *Wiki4YearOfSound2020* campaign. The Audiology Study Group participants’ contributions are presented next for each of the global campaigns. The selection of topics and subsequent literature searches were conducted by the students and their project coordinator, focusing on topics that could contribute to the promotion of hearing health. The group expanded preexisting Portuguese-language articles in Wikipedia and translated articles that had been published on the English Wikipedia (see Table 1).

The timeframe for comparing the two versions was 30 days before activities began and 30 days after they ended. Newly created articles were accessed either 30 days after their creation or when they were uploaded to the website (for those who worked on user test pages).

In addition to the contributions shown in Table 1, study participants also performed minor edits, such as adding categories to existing pages. Those edits are important because Wikipedia uses categories to align pages by thematic groups. This enables readers to browse pages related to the same topic, thus consolidating the contents on the platform while broadening users’ reading and knowledge scope. For example, the category “audiology” was added to 26 pages, increasing the number of pages with this category by about 33% [47].

**Table 1:** Details of the Brazilian students’ contributions: Wikipedia articles edited, number of words added, and/or translated * into Portuguese by the Audiology Study Group for the *Wiki4WorldHearingDay2019* and *Wiki4YearOfSound2020* campaigns.

Linked Wikipedia Pages (Portuguese Titles Translated to English)	Number of Words Added	Number of Views 30 Days before the First Edit	Date of the First Edit on the Page	Number of Views 30 Days after the Last Edit	Date of the Last Edit on the Page
Individual protection equipment	347	4476	11/08/2019	3590	07/02/2020
Disturbance of peace	389	3683	01/27/2020	5007	04/06/2020
Decibel	20	5370	12/20/2019	2799	12/20/2019
Ménière’s disease	31	1634	11/08/2019	1612	11/08/2019
Hearing test	159	1220	11/04/2019	2516	11/28/2019
Tinnitus	8734	1503	05/14/2020	1488	11/17/2020
Cochlea	573	2006	11/06/2019	1253	12/02/2019
Noise	3	2139	12/20/2019	944	12/20/2019
Cerumen	14	995	11/08/2019	1139	11/12/2019
Organ of Corti	403	986	11/08/2019	970	04/08/2020
Hyperacusis	595	445	11/08/2019	599	04/20/2020
Conductive hearing loss	627	453	11/04/2019	531	08/20/2020
Ear canal	176	454	11/26/2019	600	03/05/2020
Presbycusis	654	432	11/06/2019	346	03/05/2020
External ear	647	1013	11/08/2019	370	12/16/2019
Pain threshold *	1272	-	12/20/2019	111	12/20/2019
Psychoacoustics	1870	336	12/20/2019	238	12/20/2019
Effects of noise pollution on health	15	386	02/12/2019	1382	11/01/2019
Noise-induced hearing loss *	17,666	-	02/19/2019	343	11/29/2019
Occupational exposure limit	915	217	11/06/2019	162	12/06/2019
Ear plug	8263	535	05/18/2020	770	07/02/2020
Acoustic trauma	2812	143	11/04/2019	145	11/04/2019
Lip reading	1072	271	03/10/2020	485	03/30/2020
Hearing and speech therapist	790	220	11/21/2019	244	12/09/2019
Occupational hearing loss	99	132	03/08/2019	174	03/08/2019
Hair cells *	26,822	-	05/07/2020	121	05/07/2020
Speech banana *	333	-	11/25/2019	31	12/02/2019
Hearing protection *	11,862	-	05/16/2020	110	05/16/2020
Sensorineural hearing loss	506	310	11/07/2019	192	12/02/2019
P300 *	3068	-	12/20/2019	63	12/20/2019
Diplacusis *	10,031	-	05/05/2020	50	05/16/2020
World Hearing Day *	1178	-	11/08/2019	19	12/09/2019
Occupational risks in dentistry *	36,162	-	05/14/2020	37	05/18/2020
Noise control *	9744	-	02/20/2019	120	02/20/2019
Audiometer *	827	-	02/14/2019	52	02/14/2019
Noise dosimeter *	2120	-	03/08/2019	49	03/08/2019

Source: Outreach Dashboard and Pageview Analysis [29,48,49]; * Pages translated from English into Portuguese.

The impact of hearing health edits made by the undergraduate students of the Audiology Study Group is small (Table 1) when compared to the numbers of views of global campaigns (Table 2 and Table 3). However, the study group was responsible for 60% of the edits during the *Wiki4WorldHearingDay2019* campaign and more than 90% of the edits during the *Wiki4YearOfSound2020* campaign on the Portuguese Wikipedia.

### 3.3. Outcomes by the Brazilian Students’ Efforts as an Extension Activity: Qualitative Assessment

Table 4, Table 5 and Table 6 present an overview of the *Wiki4YearOfSound2019* and *Wiki4YearOfSound2020* campaigns based on the ORES scores for the articles on Portuguese Wikipedia within the contributions of the Audiology Study Group.

## 4. Discussion

Health promotion has been identified as a strategy to increase reach and service delivery as well as to trigger social engagement [51]. Its goal is to strengthen individuals and communities, increasing their autonomy as users of health services [52]. Wikipedia is considered an important resource for healthcare information in different contexts, one that is able to reach the public, patients, students, and professionals looking for health-related information online. Wikipedia has the mechanisms to respond to highly dynamic situations, to assess the reliability of resources, to invite collaboration, and to leverage expertise; consequently, it is becoming an important platform for health promotion [22,53,54]. Its tools and metrics make it possible to publish content that could promote the uptake of hearing health services, allowing health professionals and the public to evaluate its reach, public interest, and the potential impact of these actions within the community.

### 4.1. Analysis of Reach/Pageviews of the Global Campaigns and the Contributions from Brazilian Students

An objective of the Wikipedia campaigns described in this study was to help the general public in understanding acoustics subtopics such as environmental noise, noise control, hearing and psychoacoustics, speech, and vibration. Another objective was to increase their understanding of the importance of controlling environmental and occupational noise [55].

Undergraduate students of the Speech-Language Pathology and Audiology Program were responsible for 60% of the edits on the platforms during the *Wiki4WorldHearingDay2019* campaign and more than 90% of the edits during the *Wiki4YearOfSound2020* campaign. Their focused and ongoing participation could motivate other research groups, including Wikipedians who are interested in this topic, and possibly inspire the creation of a WikiProject in Audiology within Portuguese Wikipedia, following the guidelines of other WikiProjects [56,57,58].

### 4.2. Analysis of the Article Quality Assessment of the Brazilian Students’ Contributions as an Extension Activity

The number of article visualizations was impressive, indicating both the student’s interest in a topic as well as the interest from others who use Wikipedia. This information is valuable to researchers and health and science communicators as it allows them to plan and prioritize their communication efforts. To assess the impact of the activities, an analysis of the quality of the articles (either created or expanded) was carried out. These analyses revealed measurable improvement in the existing articles and good quality in the newly created articles.

The mean metric for the *articlequality* model for the edited articles (Table 4) rose from 1.83 to 2.5, an increase of more than 36%. The mode value rose from 1 to 2 while the median remained unchanged, suggesting variability among articles. In the articles created or translated through the online campaigns (Table 5), the mean, median, and mode values registered were 3.3, 3.5, and 4, respectively. These scores account for increased rates of 33.2%, 75%, and 100% when compared with those on the other group (Table 6).

Regarding the articles that existed before the current study (Table 4), the lower the previous quality index, the higher the increase registered. While no articles with a previous ORES quality index (*articlequality* method) of 3 or above had a change in rating after the interventions, most articles with a previous index of 2 registered a 1 point increase, and all articles with a previous score of 1 registered a 1 or 2 points increase. The pages “Tinnitus,” “Psychoacoustics,” and “Effects of noise pollution on health” had the most progress (+2) in this category. Of the 24 articles from this group, 14 evolved in terms of quality index, although 10 did not register any progress. As for pages that were created or translated from English into Portuguese (Table 5), a mode of 4 was observed. The “Hair cells” and “Hearing protection” pages achieved the highest indexes (of 5), which is equivalent to other pages retaining the “good article” status.

Although article length is often a measure to assess the quality of an article, the results demonstrated that this variable may have little influence on the article quality model predicted by ORES algorithms [59]. Other variables that impact internal quality include the quality of the text’s semantics; reliability of references; amount of inner, external, and broken links; existence of multimedia files, and more. These aspects may receive considerably more attention when an article is new [60] (pp. 101–113).

### 4.3. Feasibility of Educational Activities Involving Student Participation in Wikipedia Campaigns

While the approach described in this study is applicable to any field, it seems particularly relevant to the promotion of hearing care, as delay in seeking treatment is seen in several countries, regardless of the differences in availability of healthcare services. This delay is likely to limit the success of many interventions [1,2,3,4,5,6]. While numerous factors can explain why it takes individuals a long time to seek help, researchers argue that many of these factors are influenced by access to information regarding health conditions and the possibilities of care [1,2,3,4,5,6].

In the current study, undergraduate students were able to meaningfully contribute to the efforts of the Wikipedia component of two global health awareness campaigns. Whereas prior studies have used Wikipedia editing in the classroom as a required assignment [61,62], the current experience did not involve credit or grades [63]. Overall, the experience of student editing is described as having a positive impact [62,63].

### 4.4. Study Limitations

This study extended prior findings, but there were limitations inherent in the approach taken. Although students’ participation was not factored into grades, students may have felt that their participation would be viewed favorably by the instructor. There are many complexities involved in participatory action research, and we were unable to include them in the present study. This exploratory study, however, revealed several alternatives for follow-up work. The current study was limited in narrowly focusing on Wikipedia platforms rather than broader level technology skills. Future research should examine if this activity has a measurable impact on information literacy, such as if the skills learned through Wikipedia editing carry over to other online environments or influence other online writing, such as blogs and/or discussion forums. In addition, this approach offers opportunities for the role of collaboration in the learning process to be evaluated. Future research could (1) compare individual student work with collaborative editing to determine the effectiveness of each method, (2) strive to collect anonymous data for student feedback, and (3) allow researchers to examine whether Wikipedia activities enhance or limit student learning. An option for large classes would be to assign group work where article feedback would be given to groups rather than individual students. Regarding learning outcomes, we recommend future studies to explore the content of students’ edits to determine if the quality of their contributions improved over time.

## 5. Conclusions

Technology offers new ways for interacting and acquiring knowledge, which are extremely relevant in the health education field. Currently, skill- and knowledge-based crowdsourcing strategies are important in the promotion of public health. Awareness campaigns, such as those promoted by WHO and Wikipedia, are unique opportunities to improve hearing-related content on a referenced, plain language, open access source, thus providing quality information to the public.

The quality and the amount of coverage of science topics in Wikipedia articles are influenced by the degree in which experts in the field are involved. The expansion and improvement in articles from one subject area usually lead to an increase in pageviews [11,64]. Both approaches—translating content and improving preexistent articles—proved to be equally effective in improving the overall quality of the selected articles. These actions could contribute to the hearing health of the population by providing more freely available online articles containing reliable references as a primary source of information. Furthermore, access to plain language information about a health condition, including its possible diagnosis and treatment, is considered an important tool in increasing the use of health recommendations and services [8].

This educational and health promotion process involved study participants using new strategies to expand the delivery and implementation of scientific content. Reflecting on the study results, which indicated an average improvement of about 36% in the quality scale for the articles, incorporating activities to expand and improve scientific content in Wikipedia are justified both as a hearing health promotion educational experience and as a strategy for knowledge dissemination [65]. Clearly, Wikipedia activities provide a meaningful instructional assignment that can also benefit society.

## Figures and Tables

**Table 2:** Results from the *Wiki4WorldHearingDay2019* campaign showing Portuguese Wikipedia edits and the Audiology Study Group contributions.

	Number of Articles Created	Number of Articles Edited	Number of Words Added	Number of Article Views
Global campaign *	7	90	74,000	2,800,000
Portuguese Wikipedia (ptwiki)	6	4	43,493	13,018
Audiology Study Group	4	2	30,471	3890

Source: Provided by Outreach Dashboard and Pageviews Analysis [29,48,49]. * Includes Wikipedia website in nine languages (enwiki, eswiki, zhwiki, ptwiki, itwiki, svwiki, frwiki, zuwiki, chwiki). It is noteworthy that of the 10 pages created and edited in Portuguese, 6 were contributed by study participants.

**Table 3:** Results from the *Wiki4YearOfSound2020* campaign, based on review of global actions, Portuguese Wikipedia edits, and the Audiology Study Group contributions.

	Number of Articles Created	Number of Articles Edited	Number of Words Added	Number of Articles Viewed
Global campaign	80	1090	235,000	131,000,000
Portuguese Wikipedia (ptwiki)	4	27	26,217	766,516
Audiology Study Group	4	21	26,116	296,270

Source: Outreach Dashboard platform and Pageviews Analysis [29,48,49].

**Table 4:** Wikipedia articles (on Portuguese Wikipedia) for which an increase in quality assessment was registered following the contributions by the Audiology Study Group during the *Wiki4WorldHearingDay2019* and *Wiki4YearOfSound2020* campaigns.

Linked Wikipedia Pages (Portuguese Titles Translated to English)	Article Quality before the Interventions	Article Quality after the Last Edit on the Page	Article Quality Progress	Date of the First Edit on the Page	Date of the Last Edit on the Page
Individual protection equipment	2	3	+1	11/08/2019	07/02/2020
Disturbance of peace	1	2	+1	01/27/2020	04/06/2020
Tinnitus	2	4	+2	05/14/2020	11/17/2020
Organ of Corti	1	2	+1	11/08/2019	04/08/2020
Hyperacusis	1	2	+1	11/08/2019	04/20/2020
External ear	1	2	+1	11/08/2019	12/16/2019
Psychoacoustics	1	3	+2	12/20/2019	12/20/2019
Effects of noise pollution on health	1	3	+2	02/12/2019	11/01/2019
Occupational exposure limit	1	2	+1	11/06/2019	12/06/2019
Ear plug	2	3	+1	05/18/2020	07/02/2020
Lip reading	1	2	+1	03/10/2020	03/30/2020
Hearing and speech therapist	1	2	+1	11/21/2019	12/09/2019
Sensorineural hearing loss	1	2	+1	11/07/2019	12/02/2019

Source: Outreach Dashboard and ORES UI Toolkit [48,49,50]. Tool: ORES UI. Indexes higher than 4 should be considered as an indication of evolution and not actually representative of “good article” and/or “featured article” status, as these statuses are obtained through a community-based decision process.

**Table 5:** Quality assessment for pages translated from English into Portuguese by the Audiology Study Group during the *Wiki4WorldHearingDay2019* and *Wiki4YearOfSound2020* campaigns.

Page Title/Link to Portuguese Wikipedia	Article Quality after the Last Edit on the Page	Date on Which the Translation Was Started	Date of the Last Edit on the Page
Pain threshold	2	12/20/2019	12/20/2019
Noise-induced hearing loss	4	02/19/2019	11/29/2019
Hair cells	5	05/07/2020	05/16/2020
Speech banana	1	11/25/2019	12/02/2019
Hearing protection	5	05/16/2020	05/16/2020
P300	4	12/20/2019	12/20/2019
Diplacusis	4	05/05/2020	05/16/2020
World Hearing Day	3	11/08/2019	11/17/2020
Occupational risks in dentistry	4	05/14/2020	05/18/2020
Noise control	3	02/20/2019	02/20/2019
Audiometer	2	02/14/2019	02/15/2019
Noise dosimeter	3	03/08/2019	03/08/2019

Source: Outreach Dashboard and ORES UI Toolkit [48,49,50]. Tool: ORES UI.

**Table 6:** Analysis of quality assessment as estimated by ORES for expanded (edited) and newly created articles contributed by the Audiology Study Group on the Portuguese Wikipedia.

Articles improved (Group 1)—mean, median, and mode:				
		**Before**	**After**	**Progress**
Mean		1.83	2.5	+36.6%
Median		2	2	-
Mode		1	2	+100%
Based on Table 4.				
Articles created/translated (Group 2)—mean, median and mode:				
Mean			3.3	
Median			3.5	
Mode			4	
Based on Table 5.				
Articles created vs. articles improved:				
	**Expanded Articles (Group’s Last Edits on the Page)**	**Created/Translated Articles**	**Index of Quality for Created Content over Improved Content**	**Ratio**
Mean	2.5	3.3	0.8	+33.2%
Median	2	3.5	1.5	+75%
Mode	2	4	2	+100%

All scored metrics had relatively higher quality for translated/created articles when compared with preexisting pages. The most frequent ORES indexes (mode) within the *articlequality* method were 4 for created pages and 2 for preexistent pages. The mean and the median values were about 33% and 75% higher for the created articles compared with the expanded ones, respectively. Beyond that, an improvement was observed in the quality of 58% of the expanded articles.

## Data Availability

Only publicly available datasets were analyzed in this study. All data and logs are available publicly in Wikimedia sites and can be searched via non-proprietary software. This data can be found here: https://outreachdashboard.wmflabs.org/courses/International_Society_of_Audiology/Edit-a-Thon_for_World_Hearing_Day_2019 (accessed on 6 April 2023) and https://outreachdashboard.wmflabs.org/courses/National_Institute_for_Occupational_Safety_and_Health/Wiki4YearofSound2020 (accessed on 6 April 2023).
